# Predicting Clinical Response to Monoclonal TNF Inhibitors in Rheumatoid Arthritis: A Transcriptomic Approach Based on Transmembrane TNF Reverse Signaling and Nrf2 Activation

**DOI:** 10.3390/diagnostics15101232

**Published:** 2025-05-14

**Authors:** Katy Diallo, Yannick Degboé, Michel Baron, Anaïs Bellin-Robert, Jean-Frédéric Boyer, Adeline Ruyssen-Witrand, Arnaud Constantin, Benjamin Rauwel, Alain Cantagrel, Jean-Luc Davignon

**Affiliations:** 1 Institut Toulousain des Maladies Infectieuses et Inflammatoires (INFINITY), INSERM UMR1291, 31024 Toulouse, France; diallokaty@gmail.com (K.D.); michel.baron@inserm.fr (M.B.); anais.bellin-robert@inserm.fr (A.B.-R.); constantin.a@chu-toulouse.fr (A.C.); b.rauwel@invivogen.com (B.R.); 2 Faculty of Health, CHU Toulouse, University of Toulouse, 31059 Toulouse, France; jeanfred.boyer@gmail.com (J.-F.B.); ruyssen-witrand.a@chu-toulouse.fr (A.R.-W.); alain.cantagrel@wanadoo.fr (A.C.)

**Keywords:** rheumatoid arthritis, anti-TNF, Nrf2, reverse signaling, biotherapy, monoclonal antibodies, monocytes, prediction

## Abstract

(1) **Background**: TNF inhibitors (TNFis) have revolutionized the treatment of rheumatoid arthritis (RA). However, 30–40% of RA patients do not respond adequately to those biologics. In addition to neutralizing soluble TNF, TNFis have the ability to bind the transmembrane form of TNF, tmTNF. Importantly, tmTNF can act itself as a receptor that induces “Reverse Signaling” (RS) in cells. We previously showed that certolizumab, a Fab’ TNFi, activates RS in human primary monocytes, at least in part through the transcription factor Nrf2 that is known to regulate the expression of genes involved in anti-inflammatory response and oxidative stress. (2) **Methods**: Here, we have developed an assay for the prediction of clinical response of RA patients to TNF inhibitors. This assay is based on mRNA quantitation of CD36 activation and of six genes induced by Nrf2 following tmTNF RS in fresh monocytes. (3) **Results**: We could predict the response to anti-TNF monoclonal antibodies (mAbs) with 93.3% accuracy. However, our method was not suitable for the prediction of the response to TNF soluble receptor etanercept. (4) **Conclusions**: We have developed a rather simple, short-term test that can be standardized. Predicting the response to TNF mAbs will help physicians offer the best available treatment and provide patients with personalized medicine.

## 1. Introduction

Rheumatoid arthritis (RA) is the most prevalent chronic autoimmune inflammatory rheumatism. Its pathophysiology is largely dependent on TNF [1]. TNF inhibitors (TNFis) have revolutionized the treatment of rheumatoid arthritis (RA) [2,3]. Among TNFis, three different classes of molecules with different clinical and biological properties are currently available: three mAbs (adalimumab/ADA, infliximab/IFX, golimumab/GOLI), one soluble receptor fusion protein (etanercept/ETA), and 1 Fab’ coupled to pegol (certolizumab/CZP). Despite their high efficacy, about 30–40% of patients do not achieve an adequate response. In addition to this inter-individual variability in the response to TNFi, there is also intra-individual variability in terms of response to TNFi type [4]. To date, no theranostic test is routinely available to predict clinical response to TNFi in RA.

TNF is generated as a transmembrane precursor (tmTNF). Its cleavage by the TACE protease leads to the release of a soluble form (sTNF) which can interact with its receptor during inflammation. TNFis have the ability to bind sTNF, and consequently, they decrease the pro-inflammatory burden of TNF, by preventing its fixation on TNF receptors 1 and 2 (TNFR1/2). However, neutralization of sTNF may not be related to amelioration of the disease. The different TNFis have similar affinity for sTNF and their ability to neutralize sTNF is comparable, but the clinical response of RA patients to TNFi is not dependent on serum TNF levels [5,6].

In addition to neutralizing sTNF, TNFis have the ability to bind tmTNF. Importantly, tmTNF can act itself as a receptor that induces “Reverse Signaling” (RS) in cells [7]. We previously showed that certolizumab (CZP) activates RS in human primary monocytes, at least in part through the transcription factor Nrf2 [8]. Nrf2 regulates the expression of genes involved in anti-inflammatory response and oxidative stress [9]. Work from others highlighted that Nrf2 has a protective role against murine arthritis [10]. Our group recently demonstrated in a triple transgenic (3TG, TNFR1−/−, TNFR2−/−, and tmTNFKI/KI) mouse model that ETA and murine mAb (MP6-XT22) can induce RS and subsequently polarize macrophages towards a pro-resolutive phenotype, triggering the production of anti-inflammatory effectors and reduce arthritis [11]. We thus hypothesized that the RS of tmTNF plays an important role in the therapeutic response to TNFi in humans as well.

In the current study, we assessed whether RS and its downstream Nrf2 targets in human monocyte-derived macrophages exposed to mAbs TNFi could predict the clinical response to TNFi in RA. We were able to show that not all individuals activated Nrf2, which allowed us to define two statuses: Nrf2 activator versus non-activator. Based on these observations, we conducted a clinical study to correlate the activation status of genes of the Nrf2 pathway by RS with the clinical response of RA patients treated with TNFi, either mAbs or monoclonal receptor. We observed that the activation of the Nrf2 pathway by RS triggered by mAbs was correlated with a good clinical response. Our findings support the usefulness of such an approach to predict the clinical response to TNFi mAbs in RA.

## 2. Materials and Methods

### 2.1. Patients and Methods

#### 2.1.1. Donors

Adult patients with RA (ACR/EULAR criteria) were recruited in our department of Rheumatology (CHU Toulouse, Toulouse, France). The study protocol was approved by the Ethics committee of the Institution “CPP” sud ouest et outre mer II—ARS Midi Pyénées (DC 2016-2804), 1 December 2016. All subjects provided signed informed consent. Included patients had active disease (DAS28 > 2.6), with an indication to TNFi according to their rheumatologist. Patients receiving > 10 mg of prednisone equivalent/day within the prior 2 weeks were excluded [12,13]. Patients were included in the study prior to the initiation of the TNFi.

Samples from healthy donors (HDs) were obtained from the EFS (Etablissement Français du Sang, La Plaine Saint-Denis district, France).

Demographic and clinical data of the RA patients were collected for analysis [14].

#### 2.1.2. Cell Culture

Blood monocytes were purified from fresh blood (up to 40 mL) from RA patients or buffy coat (HDs), layered on Pancoll gradient (PanBiotech, Aidenbach, Germany) from healthy blood donors, and positively isolated by CD14+ magnetic sort following manufacturer’s instructions (Life Technologies, Carlsbad, CA, USA). Positive CD14+ sorting is widely used and was implemented in our previous paper (Degboé et al.). In our experience, this does not modify monocytes’ rate of activation.

Purified monocytes were plated and cultured at a density of 1 × 10^6^ cells/well (6-well plate, Falcon poly-styrene) in RPMI (Invitrogen, Carlsbad, CA, USA) supplemented with 10% FBS (Life Technologies, CA, USA), 1% penicillin/streptomycin (Gibco, NY, UCA) and recombinant GM-CSF (20 ng/mL, Peprotech, Cranbury, NJ, USA) for 5 days for healthy blood donors to mimic inflammatory monocytes that are found in RA and 16 h for RA patients to induce their differentiation into macrophages. Then, to induce RS, cells were stimulated with 10 µg/mL of the TNFi ADA (Abbvie, Ludwigshafen, Germany) for the monoclonal antibody class, or ETA (Pfizer, Bruxelles, Belgium) for the soluble receptor class.

#### 2.1.3. Cell Fractionation and Western Blot Analysis

Macrophages were harvested after 5 days of differentiation and stimulated as specified with TNFi. Cells were washed twice with cold PBS and centrifuged. Pellets were resuspended in 500 μL of cytop Buffer (Triton 0.25%, Tris HCl 10 mM, EDTA 5 mM, EGTA 0.5 mM and proteases inhibitor cocktail) and incubated for 3–5 min on ice. After centrifugation, supernatant was kept as the soluble part (cytoplasm) and the pellet was lysed with TNEN 250:0.1 buffer (NaCl 250 mM, TrisHCl 50 mM, EDTA 5 mM, NP40 0.1% and proteases inhibitor cocktail) for 20–30 min on ice. In addition, to recover the chromatin part from the TNEN-treated pellet, after centrifugation, supernatants were kept as the insoluble part (nucleus) and pellets were lysed in Urea 8 M for 10 min at 95 °C (chromatin part). The total amount of protein was quantified by BCA assay.

Nuclear lysates were then subjected to SDS/PAGE on 4–12% polyacrylamide gels (ThermoFisher scientific, Walham, MA, USA). After transferring on 0.22 µm nitrocellulose membrane, proteins were revealed using Nrf2 (D1Z9C, 04/2018, Cell Signaling Technology, Danvers, MA, USA) and PCNA (PC10, sc-56, Santa Cruz Biotechnologies, Paso Robles, CA, USA)-specific antibodies and anti-rabbit or anti-mouse HRP-linked polyclonal antibodies (Cell Signaling, Danvers, MA, USA). Protein revelation was performed with Chemidoc (Bio-Rad, Hercules, CA, USA) and the image was analyzed by Image-Lab5.0 software (Bio-Rad, Hercules, CA, USA).

#### 2.1.4. RNA Extraction and Quantification by RT q PCR

Messenger RNA was extracted from monocyte-derived macrophages using the High Pure RNA Isolation Kit (Roche, Basel, Switzerland) and reverse transcribed with the iScript cDNA synthesis kit (Bio-Rad, Hercules, CA, USA) according to the manufacturer’s instructions. All qPCRs were performed with SYBR green mastermix (Bio-Rad, Hercules, CA, USA). Primer pair sequences are the following:

*GAPDH*: Forward 5′-ACC CAC TCC TCC ACC TTT GAC-3′; Reverse 5′-CTG TTG CTG TAG CCA AAT TCG T-3′.

*CD36:* Forward 5′-GCA GCA ACA TTC AAG TTA AGC A-3′; Reverse 5′-GCT GCA GGA AAG AGA CTG TGT-3′.

*FBXO30:* Forward 5′-CCG GCC GAG CTG GAC TGG C-3′; Reverse 5′-TCC CTG GCT CTG GCC TGG TC-3′.

*GABARA:* Forward 5′-CCG GAC AGG GTC CCC GTG AT-3′;

Reverse 5′-TCC CCA TGT CGC GCA CAC AC-3′.

*HMOX1*: Forward 5′-GGC AGA GGG TGA TAG AAG AGG-3′; Reverse 5′-AGC TCC TGC AAC TCC TCA AA-3′.

*LBR:* Forward 5′-AGC CCC TCC TCG CAG CGT TA-3′; Reverse 5′-CCT CGG CGT CTG GAA GGG GA-3′.

*MAFG:* Forward 5′-CCC TTG TCT TGC GCC TGC CT-3′; Reverse 5′-CCG GCT CCC GCT TCA CCT TC-3′.

*OSGIN1:* Forward 5′-CTG CCT GTC AGG TCC GCT GC-3′; Reverse5′-GCG TGC TCC TTC CGG TGC TT -3′.

#### 2.1.5. Statistical Analysis

All data were analyzed with GraphPad Prism5. Normality was tested by the Agostino and Pearson test. In vitro data were analyzed with Student’s *t*-test or Mann–Whitney U-test if appropriate regarding normality. Proportions regarding categorial variables were compared usng Fischer exact test. Data are represented as mean, and a *p*-value < 0.05 (two-tailed) was considered to be statistically significant.

## 3. Results

### 3.1. TNFis Are Able to Induce Nrf2 Translocation

**Figure 1:**
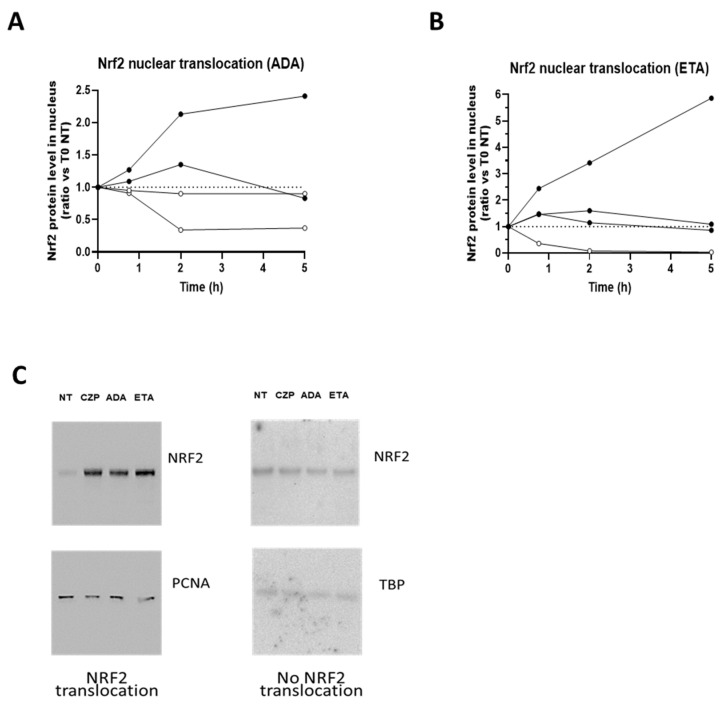
Variability of induction of Nrf2 nuclear translocation by TNFi in HD. Representative patterns are described here. Blood CD14+ monocytes purified from 4 different HDs were differentiated over 5 days into macrophages in the presence of recombinant GM-CSF and then stimulated with 10 µg/mL of TNF blockers: adalimumab (**A**) or etanercept (**B**). After 0.75, 2 or 5 h of stimulation, cells were harvested for cell fractionation. Nuclear translocation of Nrf2 was analyzed by Western blot and normalized on PCNA (Proliferating cell nuclear agent) or TBP (TATA-Box Binding Protein) expression. (**C**) Representative Western blots of Nrf2 nuclear translocation, or absence thereof, after a 5 h stimulation with CZP, ADA, or ETA.

We previously showed that the anti-inflammatory properties of TNFi were associated with Nrf2. Nrf2 transcription factor is phosphorylated and translocated into the nucleus upon activation [8]. We thus first assessed whether this activation occurred in all donors in the presence of monoclonal TNFi, ADA. HD monocytes were derived as macrophages and treated with ADA in vitro. By Western blot, we were able to confirm that stimulation of macrophages by ADA and ETA induced a nuclear translocation of Nrf2 (Figure 1A,B). Interestingly, we observed two profiles: (i) donors inducing Nrf2 nuclear translocation from 45 min, with or without maintenance of nuclear Nrf2 after 2 h, versus (ii) donors without an increase in Nrf2 nuclear translocation (Figure 1C). Only representative samples are described here.

### 3.2. Identification of Target Genes Indicating a Transcriptional Activation of tmTNF Nrf2 Signaling in Healthy Donors

**Figure 2:**
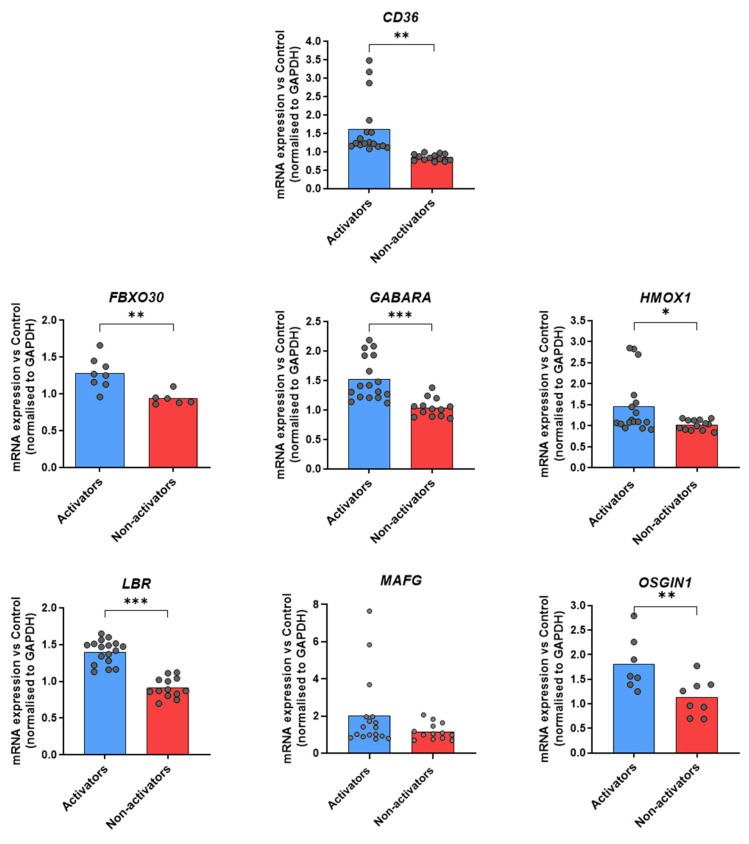
Identification of target genes indicating a transcriptional activation of tmTNF reverse signaling in HD. Global description of data obtained from HD. CD14+ monocytes purified from healthy blood donors were differentiated over 5 days into macrophages in presence of recombinant GM-CSF prior to being stimulated or not (NT) with adalimumab for 16 h for Reverse signaling activation. Cells were harvested and *cd36*, *fbx030*, *gabara*, *hmox*-1, *lbr*, *mafg* and *osgin1* mRNA expression analyzed by RT-qPCR. Donors were classified as Activators or Non-activators, based on mRNA upregulation. Data are presented as mean of mRNA fold change vs. untreated condition, normalized to gapdh. Up to 17 samples for activators and 13 for non-activators were analyzed. * *p* < 0.05, ** *p* < 0.01, *** *p* < 0.005 (paired *t*-test).

We then assessed the induction of an anti-inflammatory response upon ADA treatment, in the context of Nrf2 nuclear translocation. The rationale of the test on healthy donors was to set up methods to amplify genes of CD36 and the Nrf2 pathway and to define activators and non-activators of the pathways. To determine the stimulation time to have an optimal gene expression, we referred to our previous study on human monocytes [8]. We showed that RS activation by Fab TNFi induced scavenger receptor *CD36* and the Nrf2-target gene *HMOX1* expression. We then stimulated macrophages from HD using antibody TNFi (ADA) and analyzed *CD36* and *HMOX1* expression by RT-qPCR. According to our experience using CZP [8] and preliminary experiments, we noted a peak of gene expression after 16 h of stimulation. To go further, we analyzed the transcriptional activity of Nrf2 target genes activated by RS. We studied the modulation of fifteen Nrf2 target genes described in reference [15] by RT-qPCR in macrophages of HDs after TNFi stimulation, and selected six of them. Thus, in addition to CD36, we selected the following Nrf2 target genes (*FBX030*, *GABARA*, *HMOX1*, *LBR*, *MAFG*, *OSGIN1*), which play a role in the anti-oxidative stress response or anti-inflammatory pathway and were in our hands preferentially modulated upon TNFi stimulation. We observed two different subsets of healthy donors: donors in which TNFi stimulation increased mRNA of these target genes in macrophages (Figure 2, blue bars), and conversely, donors with no significant upregulation in transcription of these target genes (Figure 2, red bars). Based on these observations, we classified donors into two different categories: “activators” or “non-activators” of tmTNF-induced Nrf2 signaling upon antibody TNFi stimulation. However, some of the genes were not amplified (>40 cycles) in some donors, hence the discrepancies in the number of genes between donors.

We then wanted to make a link between Nrf2 transcriptional activity and its nuclear translocation. We observed that nuclear translocation of Nrf2 is not always associated with the induction of the expression of the selected target genes. Furthermore, some Nrf2 nuclear translocation profiles are not clearly delineated by Western blot. Therefore, we chose to focus on analyzing the relative amount of mRNA from Nrf2 targets (based on modulation of the expression of the six selected target genes) rather than their nuclear translocation.

### 3.3. Identification of Experimental Variation of mRNA Measurement and Definition of tmTNF Nrf2 Activator/Non-Activator Status

**Figure 3:**
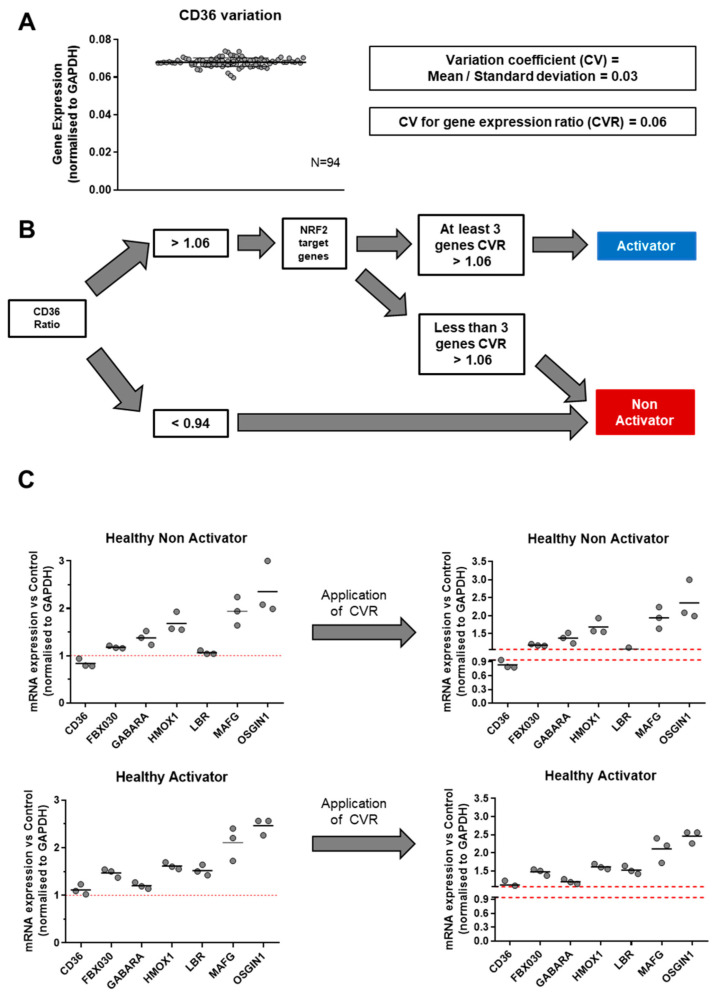
Identification of experimental variation of mRNA measurement and algorithm for the definition of tmTNF reverse signaling activator/non-activator status. (**A**) Coefficient of variation (CV) of qPCR experiments was measured by repeating the quantification of *cd36* of one sample. mRNA expression was analyzed by technical duplicate and repeated 94 times. Coefficient of variation was calculated by dividing the 94 samples’ mean by their standard deviation. (**B**) Algorithm for the definition of tm TNF Reverse signaling activator status. First of all, CD36 mRNA ratio expression (Treated/Untreated) was analyzed and after application of CVR. If this ratio was less than 0.94, individual was classified as non-activator. If CD36 ratio was greater than 1.06, an analysis of Nrf2 target genes was necessary. If at least 50% of Nrf2 target gene ratios are greater than 1.06, then individual was classified as activator. Otherwise, individual was classified as non-activator. (**C**) Example of algorithm application. Analysis of RT-qPCR replicate variability and its impact on tmTNF Reverse signaling status classification. Three replicates of RT-qPCR were performed with 1 or 2 days of interval for a healthy non-activator donor and a healthy activator donor. Application of CVR is represented by a scale cut between 0.94 and 1.06 on y axis. Red dot line corresponds the the limit of significant change according to the coefficient of variation of the test. Data are presented as mean of mRNA fold change vs. unstimulated control condition normalized to gapdh. (*n* = 3).

To optimize our definition of tmTNF-induced Nrf2 activation status, we calculated the coefficient of variation (CV) of RT-qPCR measurements. This CV was set at 0.06 because it corresponds to the highest value among all tested genes (Figure 3A).

Based on all these observations, we developed a protocol to classify donors or patients into activators or non-activators of tmTNF-induced Nrf2 after antibody TNFi stimulation. After 16 h of TNFi treatment, we first analyzed the CD36 mRNA ratio (Treated vs. Non-Treated). Whenever this ratio was lower than 0.94, the donor/patient was classified as non-activator. If this ratio was higher than 1.06, then the modulation of six Nrf2 target genes (*GABARA*, *FBXO30*, *HMOX1*, *MAFG*, *LBR*, *OSGIN1*) was analyzed. If the upregulation of at least 50% of these Nrf2 target genes was >1.06, then the donor/patient was classified as a tmTNF Nrf2 activator. If not, the donor/patient was classified as a non-activator (Figure 3B,C). Following this protocol, we analyzed the effect of our RT-qPCR machine variation on the classification. To this end, we repeated the RT-qPCR experiments three times, on different days and after frost and defrost cycles. In Figure 3C, we observed that technical variation was not biologically significant and did not impact patient/donor classification. However, it suggests that borderline data may require replicates since one of the three CD36 values fell within the 0.94–1.06 area of CV values. Finally, we tested the stability of mRNA expression over time in three HDs and one RA patient (Supplementary Figure S1). The analysis was performed at day 0 and 7 days later, in the presence of ADA. In all four donors, the activation profile remained stable over this period of time.

### 3.4. Experimental Protocol to Predict TNFi Clinical Response Depending on tmTNF Nrf2 Activator Status

We tried to link clinical response to antibody TNFi during arthritis and tmTNF Nrf2 activation status. We hypothesized that “activators” and “non-activators” of tmTNF/Nrf2 corresponded to the clinical responders and non-responders to antibody TNFi, respectively.

**Figure 4:**
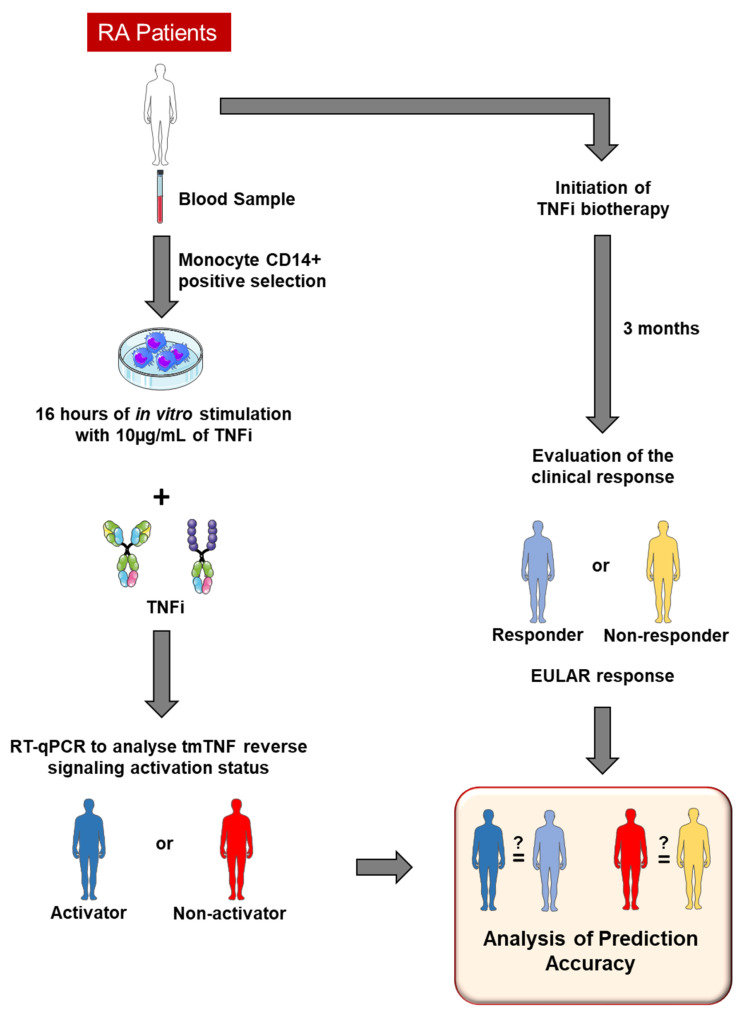
Experimental protocol to predict TNFi clinical response depending on tmTNF reverse signaling activator status. Active RA patients’ blood was collected before initiation of TNFi therapy. CD14+ monocytes were purified prior to being stimulated or not with different TNF blocker (adalimumab for the mAbs class, etanercept for the soluble receptor class) for 16 h. Cells were harvested and *cd36*, *fbx030*, *gabara*, *hmox*-1, *lbr*, *mafg* and *osgin1* mRNA expression analyzed by RT-qPCR. Depending on RT-qPCR results, patients are classified as Activators or Non-activators as a predictive marker of later clinical response. Clinical response was assessed at month 3 according to EULAR response criteria.

Then, we developed an experimental protocol to predict TNFi clinical response through the determination of tmTNF/Nrf2 activation status (Figure 4). Blood samples from RA patients were collected before the initiation of TNFi therapy. CD14+ monocytes were purified prior to being stimulated or not for 16 h with the TNFi initiated in real life (10 µg/mL antibody). Cells were harvested and Nrf2-induced genes *FBXO30*, GABARA, HMOX1, LBR, MAFG and OSGIN1 as well as CD36, which does not belong to the Nrf2 pathway, had their mRNA expression analyzed by RT-qPCR. Depending on gene modulation, patients were classified as Nrf2 Activators or Non-activators by tmTNF. A flow chart is reported in Figure 4. After 3 months, the clinical response was evaluated by physicians and patients were classified as responder or non-responder to TNFi treatment following EULAR criteria [14]. Baseline Nrf2 and CD36 activation status was compared to the real-life 3-month clinical response to assess prediction accuracy.

We included 15 RA patients treated with mAb (ADA, GOLI, IFX) and 10 patients treated with the soluble receptor ETA. Table 1 shows the clinical characteristics of patients at inclusion. Based on our activator classification, we successfully predicted the 3-month clinical response in 14/15 patients (93.3%) treated with antibodies (Table 2). Patient #2, classified as an activator, needed an increase in GOLI dose (50 to 100 mg) to achieve a satisfactory clinical response, which was thus considered as successfully predicted. On the other hand, patient #13 did not achieve a satisfactory response to GOLI, although they were predicted to be a good responder. It was the only wrong prediction in the antibody group. The prediction thus reached 93.3%. However, in patients treated with ETA, the mRNA profile did not bring any insightful information, as predictions were correct in none of the patients. Of note is that, in that series, all patients responded to the treatment. Finally, hierarchical clustering of CD36 and genes of the Nrf2 pathway was performed using using primary component analysis (Supplementary Figure S2A). One major cluster was identified. CD36 and LBR were shown to be separated from that major cluster. Supplementary Figure S2B illustrates hierarchical clustering that confirmed discrete R and NR signaling. These data suggest that tmTNF signaling is a major component of anti-TNF mAb response.

## 4. Discussion

In this work, using RS (Nrf2 and CD36 activation), we developed a test to predict therapeutic response to TNFi in RA patients. In a first approach conducted on HDs, we found that this signaling was not activated in all individuals. We therefore established criteria allowing us to define the activation status of RS based on the modulation of gene expression. In a second approach, we established a test to predict the clinical response to TNFi in patients with rheumatoid arthritis. This test has an accuracy of nearly 100% for antibodies.

Interest of prediction:

Evaluation of the benefit/risk ratio is essential for any treatment before its administration in order to ensure a good management of patients. TNFi biotherapies are expensive treatments, with adverse effects that can be serious. In the absence of drug intolerance, the evaluation of therapeutic efficacy is performed after 3 months of treatment, which in about 30% of cases results in therapeutic failure, sometimes even with a risk of worsening of the disease. Thus, the identification of biomarkers and the creation of tests to predict the response to these treatments would greatly improve patient management.

Identification of genes

From macrophages of HDs, we identified a panel of seven genes whose expression is modulated by the activation of RS in some donors by the three classes of TNFi. This observation allowed us to classify individuals into activators and non-activators of the Nrf2 pathway. These are the CD36 receptor and six Nrf2 transcription factor target genes involved in the response to oxidative and anti-inflammatory stress [10,16]. The analysis of Nrf2 target genes is all the more relevant since it has a protective role against experimental arthritis. Indeed, mice deficient for this transcription factor develop more severe arthritis compared to wild mice [10,16]. We assume that patients activating a specific gene of RS will have a good response to TNFi antibody treatment.

From a methodological point of view, the identification of Nrf2-targeted genes activated by RS was performed with healthy blood donors’ monocytes differentiated in macrophages in order to increase the expression of tmTNF. This differentiation was not necessary for RA patient monocytes. Indeed, the inflammatory environment from which they are derived results in higher tmTNF expression as compared to HDs [17]. In addition, direct testing of newly isolated monocytes from patients helps to retain the imprint of the environment in which they were found.

Several studies have been conducted to identify markers for predicting the response to TNFi in relation or not with tmTNF in rheumatoid arthritis. For example, Nguyen and colleagues showed that the monocytes of the ADA responder patients expressed more tmTNF than the non-responders and that the increase in the binding of this TNFi is a marker of a good therapeutic response for ADA but not ETA [18]. Thus, ETA seems to behave differently from ADA in terms of inducing tmTNF as well as genes induced by RS [19]. It is of note that, contrary to what has been claimed, ETA is indeed capable of inducing RS, as evidenced by our work [11]. Our data are in accordance with a different recruitment of signaling proteins in response to ADA and ETA. This dual response is reflected by accurate prediction for mAbs, but not ETA. Other work has identified CD11c expression as a marker for predicting ADA response by analyzing the transcriptome of patient blood monocytes. However, the prediction took into account only one TNFi, and in addition, it is not valid when the latter is in association with methotrexate [20]. Another study conducted by Meusch and colleagues showed that following the activation of RS of tmTNF of monocytes, increased secretion of soluble forms of TNF and IL-1 receptors (sTNFR1 and sIL-1R1) and IL-10 was correlated with a good therapeutic response to ETA in patients with rheumatoid arthritis according to EULAR criteria [21]. These discoveries, like many others, have certain limitations, such as the fact that only one TNFi has been tested, in contrast to our study, which considers the two main classes of TNFi. Unlike some studies, the prediction test we have developed is viable for patients with TNF monotherapy or in combination with other treatments (such as methotrexate). Its technicality is relatively rapid, simple and inexpensive, so the test can easily be reproduced in other laboratories with a standardized kit.

To better understand the activator/non-activator status, we need to further study the downstream signaling cascade of the tmTNF following the activation of Nrf2-induced genes and CD36 by RS.

The internalization of the TNFi found in mice BMDM following the tmTNF binding was also described in dendritic cells [22] and human macrophages in our laboratory. It would seem that this internalization is necessary for the subsequent signaling. Based on data from the literature and observations made in the laboratory, we can make some assumptions about the signaling pathways initiated.

Role of reverse signaling:

Transmembrane TNF is a signaling protein. The intracellular domain of tmTNF contains sequences of amino acids preserved between different species. In particular, it contains a consensus sequence of binding to casein kinase I (CKI) at the level of its cytoplasmic domain. Thus, on a murine macrophage line, casein kinase was shown to phosphorylate tmTNF, which is dephosphorylated following the binding of soluble TNF receptors on tmTNF [23]. In addition, the activation of RS following stimulation by the LPS activates the signaling channels of the MAP kinase and the ERK pathway [24].

Analysis of the transcriptome of macrophages following the activation of RS may bring information on genes that are differentially modulated and thus on mobilized signaling pathways that could be different depending on the TNFi. Despite the many similarities (such as the speed of association and dissociation as well as the affinity of binding to the soluble TNF of Adalimumab, Infliximab or Etanercept, which are similar (25), the differences between the TNFis both pharmacologically and functionally could justify this difference in signaling. Indeed, TNFis have a different stoichiometry; a molecule of Etanercept binds a single trimer of TNF, while this same trimer can be linked by up to three molecules of Infliximab or Adalimumab [25]. This bond difference could result in the recruitment of different proteins at the cytoplasmic level of the tmTNF and thus a different downstream signaling cascade. Indeed, we hypothesize that RS would not be limited only to the signaling of tmTNF, but rather would involve an endocytic complex with the recruitment of neighboring proteins. This could explain the difference between mAbs and ETA in predicting the therapeutic response.

The identification of genes specifically modulated by each anti-TNF through transcriptomic analysis will allow us to increase the accuracy of the test. Similarly, the comparative analysis of macrophages RNAseq from responders versus non-responders could make it possible to identify key players in this signaling that explain the differences between activators and non-activators. In the future, this test can guide clinicians in the choice of treatment to be administered in order to optimize the management of patients with rheumatoid arthritis.

Accuracy of prediction:

The test shows very satisfactory efficacy for prediction of the response to antibody but is not adequate for the other two types of TNFi, namely the soluble receptor and Fab. This reflects the various aspects and signaling pathways initiated by RS. It appears clearly from this work that the RS induced varies from one TNFi to another. The final objective of this work is to perform tests routinely before the administration of TNFi treatment so that the clinician can be guided in the choice of the TNFi to be administered. Thus, although the genes have been selected from macrophages derived from healthy blood donors, they appear to be adequate for prediction of the response of RA patients to monoclonal anti-TNF antibodies, but not to ETA. This list of genes could certainly be adapted to each class of TNFi. The cell surface levels of tmTNF on macrophages from RA patients that respond to ADA have been shown to be increased [18]. This may be related to higher RS response and better predictive response.

Possible extension to other pathologies:

TNFi are used in the treatment of pathologies other than RA. TNF is a cytokine involved in the pathophysiology of other chronic inflammatory diseases such as chronic inflammatory bowel disease (IBD), spondylitis, psoriatic arthritis, and intra- and inter-individual variability in response is also observed in these patients. Thus, we propose to evaluate the effectiveness of the prediction test in other diseases treated with TNFi in which RS and Nrf2 activation could also play a role in patient response. Indeed, it has been shown in patients with IBD that the level of tmTNF (but not that of soluble TNF) in monocytes is associated with a good response to TNFi, thus suggesting the involvement of RS [26].

Limitation of the study:

The protocol used here is suitable for the prediction of the response to mAb but not soluble TNFR2 (ETA). Reproducibility in different laboratories is required before the protocol is routinely used for prediction.

## 5. Conclusions

Signaling through tmTNF contributes to the therapeutic response to TNFi and thus constitutes a complementary mechanism of action. We also found that RS is not activated in all individuals. By testing the activation status of *NRF2* genes and CD36 by RS, we were able to develop a predictive test of the clinical response to TNFi in patients with rheumatoid arthritis. Nevertheless, this study needs to be duplicated in a separate cohort. This test could significantly improve patient management.

## Figures and Tables

**Table 1:** Characteristics of patients at inclusion. Fisher’s test for proportions and Mann–Whitney test for means. IQR: interquartile range (25th–75th percentiles).

	Monoclonal	Etanercept	*p*-Value
Women (%)	9/15 (60.0)	5/10 (50.0)	0.697
Age (IQR)	63.3 (58.0–70.0)	61.0 (55.5–67.5)	0.402
ACPA positive	14/15 (93.3)	9/10 (90.0)	0.999
RF positive	13/15 (86.7)	8/9 (88,9)	0.999
Erosion	6/15 (40.0)	6/10 (60.0)	0.428
Baseline DAS28 (IQR)	4.82 (4.15–5.58)	4.60 (3.91–5.31)	0.521

**Table 2:** Evaluation of the predictive performance in RA patients. Disease activity (DAS28-CRP), clinical response at 3 months and prediction test for each individual treated with TNF blocker (etanercept/ETA, golimumab/GOLI, adalimumab/ADA or infliximab/IFX). Prediction of response: « + » relates to « activator » of NRF2 genes response, « − » relates to « non-activator » thus to absence of response of NRF2 genes. Clinical evolution: « + » relates to positive clinical response, « − » relates to absence of significant clinical response. “?” relates to uncertain test. Prediction is considered as good (green), wrong (red) or uncertain (purple).

Molecule	DAS28 M0	DAS28 M3	Prediction of Response	Clinical Evolution	Predictive Value
**ANTIBODIES**					
GOL	5.09	4.47	−	−	
GOL	4.50	2.00	+	+	
ADA	5.40	3.10	+	+	
GOL	4.58	2.35	+	+	
ADA	3.78	1.04	+	+	
ADA	4.79	1.21	+	+	
ADA	7.10	5.16	−	−	
GOL	4.15	2.94	+	+	
IFX	4.39	2.61	+	+	
ADA	6.02	5.37	−	−	
IFX	5.60	3.17	−	−	
ADA	2.62	1.40	+	+	
GOL	3.78	4.16	+	−	
ADA	4.92	3.04	+	+	
ADA	5.58	3.15	+	+	
**ETANERCEPT**					
ETA	3.72	1.73	−	+	
ETA	5.88	2.50	−	+	
ETA	4.55	2.25	−	+	
ETA	4.64	2.56	−	+	
ETA	5.12	2.59	+?	+	
ETA	3.55	2.69	−	+	
ETA	4.63	2.82	+?	+	
ETA	6.03	1.40	−	+	
ETA	3.97	1.50	+	+	
ETA	4.18	2.12	+	+	

## Data Availability

The data presented in this study are available on reasonable request to the corresponding authors.

## Supplementary Materials

The following supporting information can be downloaded at: https://www.mdpi.com/article/10.3390/diagnostics15101232/s1. Supplementary Figure S1: Stability of mRNA expression over time. Supplementary Figure S2: Hierarchical clustering according to gene panel and clinical response.
