# Supplementary material for: Predicting Clinical Response to Monoclonal TNF Inhibitors in Rheumatoid Arthritis: A Transcriptomic Approach Based on Transmembrane TNF Reverse Signaling and Nrf2 Activation
Source: Diagnostics (Basel). 2025 May 14;15(10):1232. doi: 10.3390/diagnostics15101232 (PMC12109967; doi:10.3390/diagnostics15101232)

Supplementary Figure S1

A

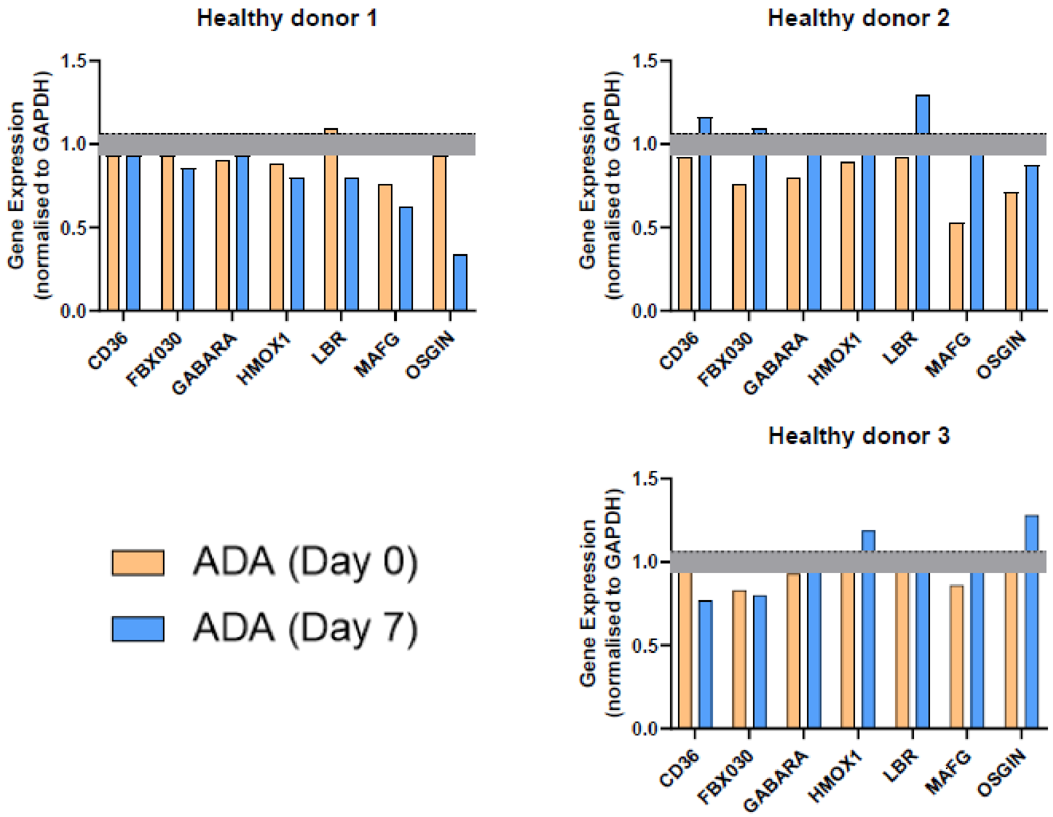

B

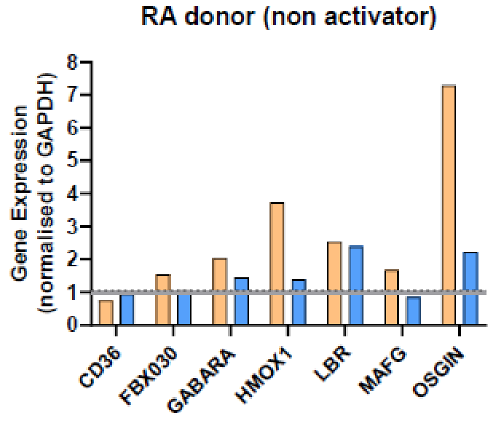

Suppl Figure S1: Stability of the mRNA profiles 3 healthy donors (A) and 1 RA patient (B) had mRNA profile analysis at day 0 and 7 days later to assess the stability of the profile. In this 4 donors, the activation profile remained stable in this period of time.

Supplementary Figure S2

A

Hierarchical clustering was performed. It does show that there is a major cluster of genes and that CD36 and LBR are clearly separated to each other and do not belong to the major cluster. Proportion of variance: PC1 46.57%, PC2 19.45%.

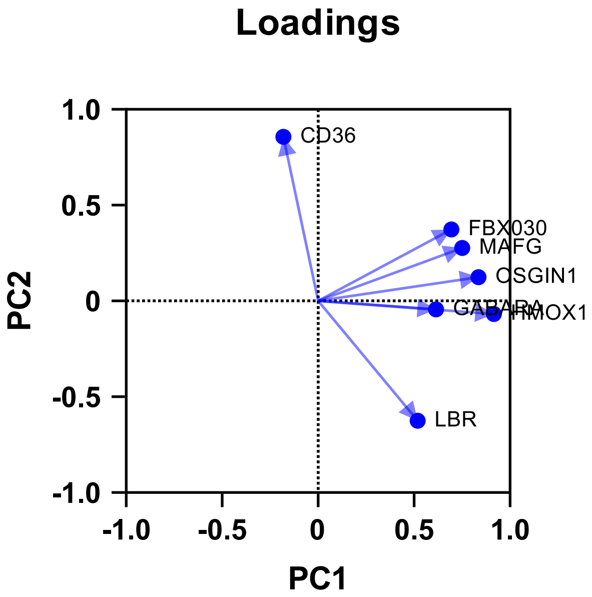

B

Responders (R) and Non Responders (NR) belonged to separate clusters.

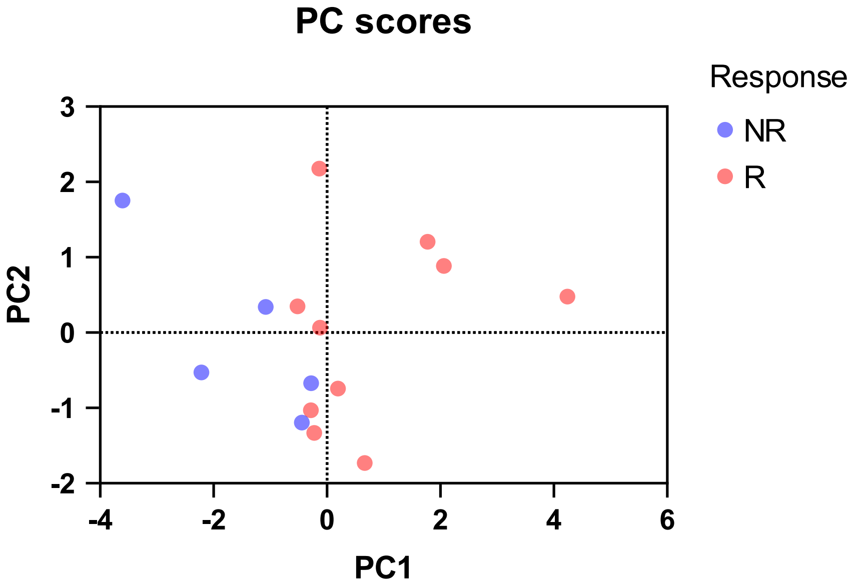

Supplement: Supplementary file 1 [file diagnostics-15-01232-s001.zip › diagnostics-3631495-supplementary.pdf]
